# N-of-1 Trials: Evidence-Based Clinical Care or Medical Research that Requires IRB Approval? A Practical Flowchart Based on an Ethical Framework

**DOI:** 10.3390/healthcare8010049

**Published:** 2020-02-27

**Authors:** Bas C. Stunnenberg, Jaap Deinum, Tom Nijenhuis, Frans Huysmans, Gert Jan van der Wilt, Baziel G.M. van Engelen, Frans van Agt

**Affiliations:** 1 Department of Neurology, Donders Institute for Brain, Cognition and Behaviour, Donders Center for Medical Neuroscience, Radboud University Medical Center, 6500 HB Nijmegen, The Netherlands; Baziel.vanEngelen@radboudumc.nl; 2 Department of Internal Medicine, Radboud University Medical Center, 6500 HB Nijmegen, The Netherlands; jaap.deinum@radboudumc.nl; 3 Department of Nephrology, Radboud University Medical Center, 6500 HB Nijmegen, The Netherlands; Tom.Nijenhuis@Radboudumc.nl; 4 Commissie Mensgebonden Onderzoek, Research Ethics Committee, 6500 HB Nijmegen, The Netherlands; Frans.Huysmans@radboudumc.nl (F.H.); Frans.vanAgt@radboudumc.nl (F.v.A.); 5 Department of Health Evidence, Radboud University Medical Center, 6500 HB Nijmegen, The Netherlands; GertJan.vanderwilt@radboudumc.nl

**Keywords:** N-of-1 trial, single patient trial, ethics, institutional review board

## Abstract

N-of-1 trials can provide high-class evidence on drug treatment effectiveness at the individual patient level and have been given renewed interest over the past decade due to improvements of the initial single patient design. Despite these recent developments, there is still no consensus under what circumstances N-of-1 trials should be considered as part of evidence-based clinical care and when they represent medical research with need for institutional review board (IRB) approval. This lack of consensus forms an obstacle for a more widespread implementation of N-of-1 trials. Based upon the existing literature, we as a group of researchers involved in N-of-1 trials and members of the IRB of a tertiary academic referral center, designed a practical flowchart based on an ethical framework to help make this distinction. The ethical framework together with a practical flowchart are presented in this communication.

## 1. Introduction

N-of-1 trials (i.e. multiple cross-over, double-blind, placebo-controlled single patient trials) can provide high-class evidence at the single patient level regarding drug treatment effectiveness or causality of side effects in chronic conditions [1]. Over the past decades, the N-of-1 trial design has received renewed interest due to improvements of the initial single patient design, such as an aggregated N-of-1 trials design with Bayesian approach [2] to provide evidence on the effectiveness of a drug at the single patient and group level simultaneously [3]. This is of particular interest in the context of building the evidence-base for treatments that can be considered for patients with rare diseases in which large randomized controlled trials are hampered by low patient numbers and large clinical and genetic heterogeneity [4].

Despite these recent developments in the design and statistical analysis of (multiple) N-of-1 trials, the debate on the ethical framework of N-of-1 trials is unresolved and remains an obstacle for a more widespread implementation of N-of-1 trials in clinical practice and research. This debate evolves around the question: “Under what circumstances should N-of-1 trials be considered as a method to improve individual patient clinical care in an evidence-based manner, versus medical research using N-of-1 trials which necessitates institutional review board (IRB) approval?”.

Although there have been several published viewpoints, guidelines and protocols from experienced N-of-1 trialists providing argumentation to answer these questions [5,6,7,8,9], a recent study by Cen et al. showed this has not led to a shared vision or availability of a shared protocol on this topic among IRBs in the United States [10]. Almost half (43.2%) of the 59 responding IRBs regarded N-of-1 trials as meeting the definition of research with requirement of IRB approval in a majority of responders. Both the potential of generating “generalizable knowledge” and the “systematic” nature of the investigations in an N-of-1 trial were frequently reported arguments in support of this view. No specific arguments were reported to support the view of the remaining percentage of IRBs that considered N-of-1 trials as (an extension of) clinical care (22%) without need for IRB approval, were unsure (15.9%) or made their decision on a case-by-case basis (15.9%) [10].

Based upon the literature, we (a group of researchers involved in N-of-1 trials and members of the IRB of a tertiary academic referral center) designed an ethical framework and a practical flowchart to help distinguish when an N-of-1 trial should be considered as evidence-based clinical care or as medical research that requires IRB approval, specified for different circumstances. The ethical framework and resulting practical flowchart to help decide if an N-of-1 trial should be reviewed by an IRB are presented in this communication with explanation of the content and the realization process. 

## 2. Methods

### 2.1. Consensus Meetings

Three consensus meetings were scheduled between the authors, who are all working at the Radboud University Medical Center, Nijmegen, the Netherlands, a tertiary academic referral center. Apart from clinicians involved in the conduct of N-of-1 trials (BCS, JD, TN, GJvdW, BvE), two members of the IRB (FvA and FH) took part in the meetings. In a first meeting (March 2015), the background literature on the existing ethical framework on N-of-1 trials was discussed and a first draft of the flowchart was presented (by BCS). In the second meeting, a first draft for the ethical framework was presented (by FvA) and discussed. In the third meeting, the final draft of the local protocol (that served as the base for this Communication) was discussed and group agreement was reached on the used terminology (explained below) and the full protocol. The ethical framework and practical flowchart were subsequently implemented as a local guideline in our center in April 2017. 

### 2.2. Terminology

(Individual) N-of-1 trial

We refer to an N-of-1 trial (i.e. N = 1 trial, single patient trial) as a trial in which a single patient is attributed to multiple treatment sets of active treatment and placebo treatment ideally in a blinded and randomized fashion, while measuring key symptoms, signs or lab results as outcome measure, until treatment efficacy is demonstrated or excluded [1,2,3,4,5,6,7,8,9,10,11]. Data analysis only takes place at the level of a single patient.

A series of N-of-1 trials without aggregation

In a series of N-of-1 trials (i.e. combined N-of-1 trials), multiple N-of-1 trials are carried out in a group of patients (one N-of-1 trial for each patient). In a series of N-of-1 trials without aggregation, statistical analysis only takes place at the individual levels [11]. The focus remains on the individual outcomes. Descriptive statistics on group outcomes (such as in what percentage of N-of-1 trials is the individual N-of-1 trial considered a success or resulted in a change of management after trial completion) can still be considered as part of a series of N-of-1 trials without aggregation. 

An aggregated series of N-of-1 trials

When, in a series of individual N-of-1 trials, statistical analysis is performed both at the individual and (sub)group level(s), with the goal to provide treatment estimates on these different levels, this is referred to as an aggregated series of N-of-1 trials [2,3,4,5,6,7,8,9,10,11]. Here, the goal is not only to provide high-class evidence for one or multiple patients at the individual level, but also to gain generalizable knowledge on the group-effectiveness, heterogeneity of treatment effect and factors that influence treatment response by aggregation of results.

## 3. Results

### 3.1. Ethical Framework

Before the ethical framework of N-of-1 trials is substantiated, we first introduce two more familial scenarios below, namely ‘treating one patient with an experimental treatment in regular clinical practice’ (scenario 1) and ‘medical scientific research of an experimental treatment in one patient’ (scenario 2).

#### 3.1.1. Scenario 1: Treating One Patient with an Experimental Treatment 

Experimental treatment can be defined as the process in which a doctor treats his or her patient with a treatment that has not (yet) been scientifically proven to be effective and safe (for the group of patients that the individual patient belongs to) and is furthermore not (yet) commonly used in clinical practice for the treatment of patients with similar characteristics. A doctor is allowed to start the experimental treatment in his or her patient if there are no regular, alternative treatments (remaining), the need for treatment is urgent and there is enough circumstantial evidence to expect effectiveness and safety of this particular experimental treatment for this particular patient. The experimental treatment is primarily focused on treating the (symptoms of the) disease of this one patient in regular clinical care. Nonetheless, medical information that has been gathered on the experimental treatment of this one patient can be published as a case report. Essentially, the same standards apply to the experimental treatment of patients as in the regular treatment of patients. The treating physician must be able to defend his or her choice for the experimental treatment among his colleagues and the treatment must be conducted in accordance with the generally accepted standards (for example, this means that it is not allowed to expose patients to unnecessary and potentially harmful measures to determine the treatment response). The physician is obliged to inform the patient on the experimental nature of the proposed treatment and it is advised to provide the patient also with written information and get informed consent for the experimental treatment. Approval from an IRB is however not obligatory.

#### 3.1.2. Scenario 2: Medical Scientific Research of an Experimental Treatment in One Patient 

In medical scientific research of an experimental treatment, a physician (or researcher) exposes a patient (whether or not in a double blind, randomized, placebo-controlled fashion) to an experimental treatment with the goal to answer a scientific question involving that particular treatment. The participating patient can, in a way, be seen as a mean to achieve a goal that lies outside of his or her personal level, namely to answer a scientific question. This does not imply that it is not possible for a participating patient to experience substantial benefits from participation in such a medical scientific research study, as is often the case. In a scientific research project, the choice for the treatment, dosage scheme and interval of treatment is not primarily based on what is the best treatment strategy for the participating patient individually. Medical scientific research that involves treatment of patients is covered by the ‘Medical Research Involving Human Subject Act’ (i.e., in The Netherlands ‘Wet Medisch-Wetenschappelijk Onderzoek’ (WMO)) and equivalent acts worldwide. This act requires, in all cases, a written informed consent and prior approval of the research protocol by an accredited IRB on research involving human subjects with review authority in the name of the WMO.

#### 3.1.3. Under What Circumstances Should N-of-1 Trials Be Considered as Clinical Care or Medical Research That Requires Ethical Committee Approval?

**Figure 1:**
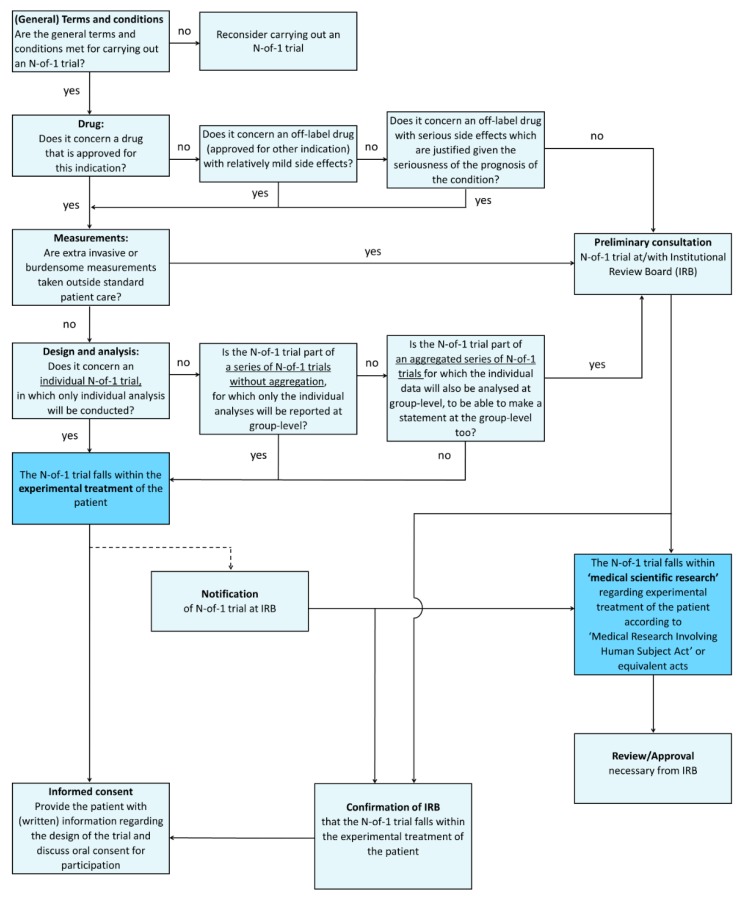
Practical flowchart to help distinguish when an N-of-1 trial should be considered as evidence-based clinical care or as medical research that requires IRB approval, specified for different circumstances.

Based upon the aforementioned ethical framework, we advise that N-of-1 trials can be considered as part of the ethical and legal framework of ‘*Treating one patient with an experimental treatment*’ (described above under scenario 1), do not require formal and integral IRB approval before the start of the trial, if the following circumstances are met:

(1) The drug is approved or prescribed off-label with relatively mild side effects, or, is an off-label drug with serious side effects whose use is justified given the seriousness of the prognosis of the condition.
(2) No additional invasive or burdensome measurements are performed outside of standard patient care.
(3) Analysis takes place on the individual level only, or when the individual N-of-1 trial is part of a series of N-of-1 trials, only individual results of statistical analysis will be reported. If the abovementioned circumstances are not met, we advise a formal and integral review and approval by the IRB (see Figure 1 for a practical flowchart that can guide in this decision) before the start of the N-of-1 trial.

It is conceivable that a physician desires a second opinion on a planned N-of-1 trial, even though the N-of-1 trial does not require an official IRB approval according to the criteria described above. For example, in case the physician has doubts about the therapeutic justification or need of the trial, or because of the need to have an independent and judicial verdict on the need of approval from an IRB (some scientific journals require such a statement upon publication). In our opinion, the key question the IRB should answer under these circumstances is whether the proposed N-of-1 trial (including choice for treatments and outcome measures), can indeed be proposed to the patient as an improvement of his or her care. If so, a formal review is not advised. The IRB can however advise on the need for a written informed consent and on its content. If the N-of-1 trial cannot be justified as an improvement of the individual patient’s care, the N-of-1 trial will need a formal and integral review process by the IRB.

## 4. Discussion

The hereby presented ethical framework and practical flowchart are designed to contribute to a broader consensus between physicians and IRBs on the need for IRB approval for N-of-1 trials, specified for different circumstances.

In our ethical framework, N-of-1 trials are not considered a medical research experiment just because of the fact that key methodological elements of clinical randomized controlled trials (e.g., blinded study medication, the use of placebo, randomization, etc.) to reduce sources of bias are incorporated in the design or systematic data collection and analysis takes place. After all, with all the sources of bias that are introduced in our generally accepted everyday ‘trials of therapy’ (in which a patient receives a drug over a certain time period, often without standardized measures in between, and reports back the subjective treatment effect at a follow-up visit) it could even be argued that this standard care is in fact unethical [1]. Instead, the question whether the N-of-1 trial really aims to improve the patient’s care, without additional invasive or burdensome measures outside regular clinical practice, plays a central role in the framework. Thus, using an N-of-1 trial design to get reliable data on treatment efficacy in one or multiple single patients (with an individual N-of-1 trial or a series of N-of-1 trials design without aggregation) can, under certain circumstances, be considered as an evidence-based improvement of care and not a medical scientific experiment. Obtaining any form of statistical generalizable evidence on drug effectiveness on the patient group level using a series of aggregated N-of-1 trials design is however, categorized as a medical scientific experiment in our framework.

The presented framework and flowchart were implemented as a local guideline in our tertiary academic referral center in 2017. It provides both clinicians and the IRB with the different scenarios upfront, in which time and effort for IRB approval are invested efficiently. Both before and after implementation, N-of-1 trials were conducted in our center [3,12,13,14]. As example, one series of N-of-1 trials without aggregation, in which the efficacy of different magnesium salts to reduce fatigue and neuromuscular symptoms in four patients with renal magnesium wasting was tested using questionnaires and lab results as outcome measures, for which IRB approval was sought, in retrospect would not have needed formal IRB approval based on the presented framework and flowchart [12]. We encourage readers to use this communication to start a dialogue within their own medical center on this topic.
